# The Association of Hypertensive Disorders of Pregnancy with Infant Mortality, Preterm Delivery, and Small for Gestational Age

**DOI:** 10.3390/healthcare12050597

**Published:** 2024-03-06

**Authors:** Dulaney A. Wilson, Julio Mateus, Emily Ash, Tanya N. Turan, Kelly J. Hunt, Angela M. Malek

**Affiliations:** 1 Department of Public Health Sciences, Medical University of South Carolina, Charleston, SC 29425, USA; eash0802@gmail.com (E.A.); huntke@musc.edu (K.J.H.); malek@musc.edu (A.M.M.); 2 Department of Obstetrics & Gynecology, Maternal-Fetal Medicine Division, Atrium Health, Charlotte, NC 28204, USA; 3 Department of Neurology, Medical University of South Carolina, Charleston, SC 29425, USA

**Keywords:** hypertensive disorders of pregnancy, eclampsia, preeclampsia, chronic hypertension, infant mortality, preterm delivery, small for gestational age, maternal and infant outcomes, racial and ethnic differences

## Abstract

Gestational hypertension, preeclampsia, eclampsia, and chronic hypertension (CHTN) are associated with adverse infant outcomes and disproportionately affect minoritized race/ethnicity groups. We evaluated the relationships between hypertensive disorders of pregnancy (HDP) and/or CHTN with infant mortality, preterm delivery (PTD), and small for gestational age (SGA) in a statewide cohort with a diverse racial/ethnic population. All live, singleton deliveries in South Carolina (2004–2016) to mothers aged 12–49 were evaluated for adverse outcomes: infant mortality, PTD (20 to less than <37 weeks) and SGA (<10th birthweight-for-gestational-age percentile). Logistic regression models adjusted for sociodemographic, behavioral, and clinical characteristics. In 666,905 deliveries, mothers had superimposed preeclampsia (HDP + CHTN; 1.0%), HDP alone (8.0%), CHTN alone (1.8%), or no hypertension (89.1%). Infant mortality risk was significantly higher in deliveries to women with superimposed preeclampsia, HDP, and CHTN compared with no hypertension (relative risk [RR] = 1.79, 1.39, and 1.48, respectively). After accounting for differing risk by race/ethnicity, deliveries to women with HDP and/or CHTN were more likely to result in PTD (RRs ranged from 3.14 to 5.25) or SGA (RRs ranged from 1.67 to 3.64). As CHTN, HDP and superimposed preeclampsia confer higher risk of adverse outcomes, prevention efforts should involve encouraging and supporting mothers in mitigating modifiable cardiovascular risk factors.

## 1. Introduction

Hypertensive disorders of pregnancy (HDP) including eclampsia, preeclampsia, and gestational hypertension are significant risk factors for adverse pregnancy outcomes such as preterm delivery (PTD), small for gestational age (SGA), and infant mortality [1,2,3,4,5,6,7,8]. The prevalence of HDP in the United States (U.S.) has been increasing over the past few decades from 2.9% in 1989 to 7.2% in 2018 [9]. 

Many risk factors for HDP overlap with those for PTD and SGA, including demographic characteristics (age, race, ethnicity, etc.) and clinical factors (pre-existing conditions such as chronic hypertension, body mass index [BMI], etc.) [1,5,6,10]. Women with pre-existing (chronic) hypertension are more likely to have superimposed preeclampsia and adverse pregnancy outcomes [11,12]. As with HDP, the prevalence of chronic hypertension has been rising over the last few decades [9]. Both PTD and SGA increase the risk of infant mortality and morbidity as well as the risk of delayed neurodevelopment that may have long-term consequences such as impaired cognition, learning difficulties, cerebral palsy, impaired hearing, and impaired vision [5,6,7]. The combination of PTD and SGA confers an increased risk of infant mortality [13]. Rates of HDP, PTD, SGA births, and infant mortality are consistently higher among Black mothers compared to White mothers [5,14,15,16,17].

Overall, there is a gap in the literature in the area of combined risk factors for infant mortality, PTD, and SGA. Most studies have considered the impact of HDP and/or eclampsia, preeclampsia, or chronic hypertension on perinatal outcomes, but few considered the combined effect of HDP superimposed on chronic hypertension. Additionally, while many studies have not considered racial or ethnic differences in regard to this exposure-outcome relationship, a number of studies have shown that racial health inequities impact infant outcomes [5,6,16,17].

We aimed to assess the relationships between HDP, superimposed preeclampsia, and chronic hypertension with infant mortality, PTD, and SGA across race/ethnic groups. Given the long-term impact of PTD and SGA on infant morbidity and mortality, identifying relevant maternal demographic and clinical risk factors may provide insight into the best care for women with HDP or chronic hypertension. 

## 2. Materials and Methods

### 2.1. Cohort Selection

This retrospective cohort study encompasses all live, singleton deliveries that occurred in hospitals in South Carolina (SC) from 2004 to 2016 to women aged 12–49 years. The analytic cohort was based on comprehensive statewide administrative billing data on individual encounters for the mother and her baby that included inpatient discharges and other outpatient services, emergency department (ED) visits, and Medicaid that was obtained from the SC Revenue and Fiscal Affairs Office (RFA), Health and Demographics Section. Vital records were obtained from the SC Department of Health and Environmental Control Vital Records Division that included birth certificate data and death certificate data for the mother and the baby. The vital statistics data were augmented with the maternal procedure and diagnosis codes from delivery with up to 14 years of follow-up available. The data are available for research purposes with permission in accordance with the policies of the respective data owners. Maternal hospitalizations for deliveries and birth certificates were successfully matched using a unique identifier from the SC RFA Office for 97.5% of the cohort. The study was approved by the Medical University of South Carolina Institutional Review Board. 

**Figure 1:**
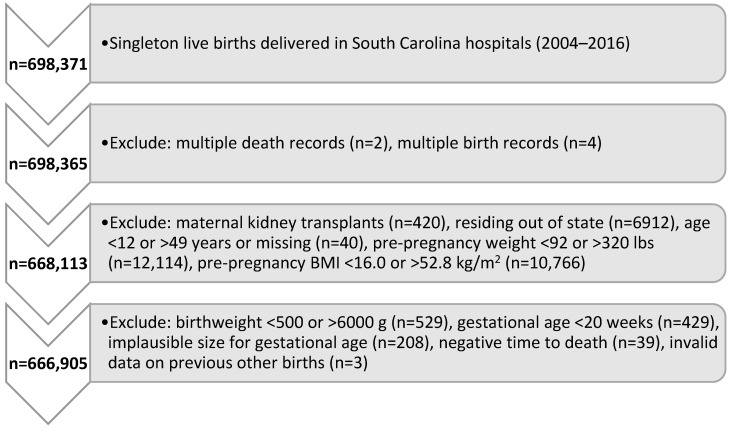
Study flow diagram showing inclusion and exclusion criteria. BMI indicates body mass index.

Between 2004–2016, there were a total of 698,371 live, singleton births in SC identified from birth certificate data with matching hospitalization/ED visit data. Deliveries were excluded if there were multiple birth or death records, mothers with prior transplants, mothers residing outside of SC, gestational age <20 weeks, and biologically implausible values for age, pre-pregnancy weight or BMI, birthweight, time to death, or invalid data on past births. The dataset for analysis consisted of 666,905 live, singleton births to 444,457 women (see Figure 1).

### 2.2. Exposures and Outcomes

The exposures, outcomes, and covariates were identified using the International Classification of Diseases, Ninth and Tenth Revision, Clinical Modification (ICD-9-CM/ICD-10-CM) diagnosis and procedure codes from hospitalization/ED visit data (Supplemental Table S1). HDP (preeclampsia, eclampsia, gestational hypertension) was defined by hospitalization/ED visit data (ICD-9/10-CM codes: 642.3–642.7, O11–O16), or HDP on the birth certificate (indicated by a gestational hypertension checkbox). Chronic hypertension was defined by hospitalization/ED visit data or birth certificate data (indicated by a pre-pregnancy hypertension checkbox). Mothers at delivery were classified into four exposure groups based on the presence of HDP and chronic hypertension: (1) chronic hypertension with superimposed preeclampsia (referred to as ‘superimposed preeclampsia’), (2) HDP without chronic hypertension (referred to as ‘HDP’), (3) chronic hypertension without superimposed preeclampsia (referred to as ‘chronic hypertension’), and (4) neither HDP nor chronic hypertension (referred to as ‘without hypertension’). 

PTD and SGA were defined by birth certificate data. PTD (20 to <37 weeks gestation) was further categorized as early PTD (20 to <34 weeks) and late PTD (34 to <37 weeks). Birthweight-for-gestational-age percentiles were calculated based on estimates from Talge et al. and infants were categorized as large-for-gestational age (>90th birthweight-for-gestational-age percentile) or SGA (<10th birthweight-for-gestational-age percentile) [18]. Infant mortality within one year of birth was defined by death certificate data. Maternal demographic and clinical characteristics obtained from the birth certificate data included age, self-reported race/ethnicity, education, income, primary payer, eligibility for Women, Infants and Children (WIC) benefits in pregnancy, smoking during pregnancy, BMI (pre-pregnancy), diabetes mellitus (pre-pregnancy and gestational), prenatal care information, number of previous pregnancies, previous cesarean delivery (C-section), previous PTD, gestational age at delivery, size for gestational age, Revised-Graduated Prenatal Care Utilization (R-GINDEX), baby sex, mode of delivery (cesarean-section or vaginal [forceps, spontaneous, vacuum]), and induced labor. Maternal race was provided as White, Black, Other and Unknown; and ethnicity was provided as Hispanic, Non-Hispanic or Unknown. Race/ethnicity was further defined as non-Hispanic White (NHW), non-Hispanic Black (NHB), Hispanic, and other. The R-GINDEX was used to measure the adequacy and utilization of prenatal care based on the trimester and gestational age when care began and the total number of prenatal care visits [19,20]. The R-GINDEX categories are as follow: inadequate, intermediate, adequate, intensive, no care, and not able to calculate due to missing information. However, the quality of health care received is not factored into the R-GINDEX. 

### 2.3. Statistical Analysis

Group differences for continuous variables were analyzed with analysis of variance (ANOVA). Categorical variables were analyzed with a Chi-square test. The associations between pregnancy and delivery characteristics including HDP, superimposed preeclampsia, and chronic hypertension with outcomes such as infant mortality within one year of birth were estimated by relative risks (RRs) and 95% confidence intervals (CIs) using logistic regression models. 

Potential interactions of exposure and race/ethnicity were evaluated for each outcome with the addition of an interaction term to the model; if the interaction was significant, the RR was reported in a joint analysis allowing the baseline risk to differ by race/ethnicity. Covariates evaluated for adjustment in modeling the outcomes included the following sociodemographic, behavioral, and clinical characteristics: maternal age (continuous), race/ethnic group, education, rural residence, income (categorical), primary payer during pregnancy, WIC eligibility during pregnancy, smoking during pregnancy, pre-pregnancy BMI (continuous), number of previous pregnancies, previous cesarean section, previous PTD, and baby sex. Models for PTD further adjusted for size for gestational age (adequate, small, and large). Post-hoc sensitivity analyses were performed to determine if deliveries to women aged 12 to 19 years significantly biased the risk estimates. Although the models were adjusted for maternal age, adolescent pregnancy is a known risk factor for infant mortality, PTD, and SGA [21]. The analyses were repeated for adults only and the change in risk estimates quantified. A change of less than 10% was considered non-significant. The analyses were performed using SAS Version 9.4 (SAS Institute Inc., Cary, NC, USA) [22].

## 3. Results

The 666,905 deliveries occurring between 2004 and 2016 were among women diagnosed with chronic hypertension (1.8%), HDP (8.0%), and superimposed preeclampsia (1.0%), or without hypertension (89.1%) (see Table 1). Overall, deliveries to mothers with chronic hypertension, HDP, and superimposed preeclampsia were more likely to result in infant mortality, PTD, or SGA than deliveries with no hypertension. Compared to deliveries with no hypertension, deliveries with chronic hypertension, HDP, and superimposed preeclampsia showed higher proportions of NHB women, and were more likely to be eligible for WIC in pregnancy and to have a higher pre-pregnancy BMI. They were also more likely to have a previous PTD, intensive prenatal care as measured by R-GINDEX, and diabetes defined as either pre-pregnancy or gestational diabetes mellitus (GDM). 

### 3.1. Association of Sociodemographic and Clinical Characteristics including Pregnancy and Delivery Complications with Infant Mortality

Table 2 presents the RRs for infant mortality adjusted for sociodemographic and clinical characteristics. Following adjustment for demographic and clinical characteristics, infants born to women with superimposed preeclampsia (RR = 1.79, 95% CI: 1.31–2.43), HDP (RR = 1.39, 95% CI: 1.21–1.58), and chronic hypertension (RR = 1.48, 95% CI: 1.16–1.88) had increased risk of infant mortality than those born to women with no hypertension. Higher infant mortality risks were also seen in NHB mothers (RR = 1.43, 95% CI: 1.30–1.57) and those with less education than a college degree (RRs ranged from 1.29 [some college] to 1.53 [less than high school]) relative to those with a college education or above; with Medicaid, self-pay, or other insurance (vs. private insurance, RRs ranged from 1.34 to 1.68); who smoked during pregnancy (RR = 1.88, 95% CI: 1.69–2.09); and for each unit increase in pre-pregnancy BMI (RR = 1.01, 95% CI: 1.01–1.02); or a previous PTD (RR = 1.94, 95% CI: 1.64–2.30) after adjustment for covariates. The adjusted risks of infant mortality were lower for each year increase in maternal age (RR = 0.98, 95% CI: 0.97–0.99), Hispanic relative to NHW mothers (RR = 0.66, 95% CI: 0.54–0.82), women with WIC eligibility during pregnancy (RR = 0.81, 95% CI: 0.73–0.89), and female relative to male sex of baby (RR = 0.75, 95% CI: 0.69–0.81). The addition of an interaction term (exposure group and race/ethnicity) to the model did not significantly affect the model (*p* = 0.65).

### 3.2. Joint Analysis of Race/Ethnicity, Chronic Hypertension, HDP and Superimposed Preeclampsia with PTD and SGA

**Figure 2:**
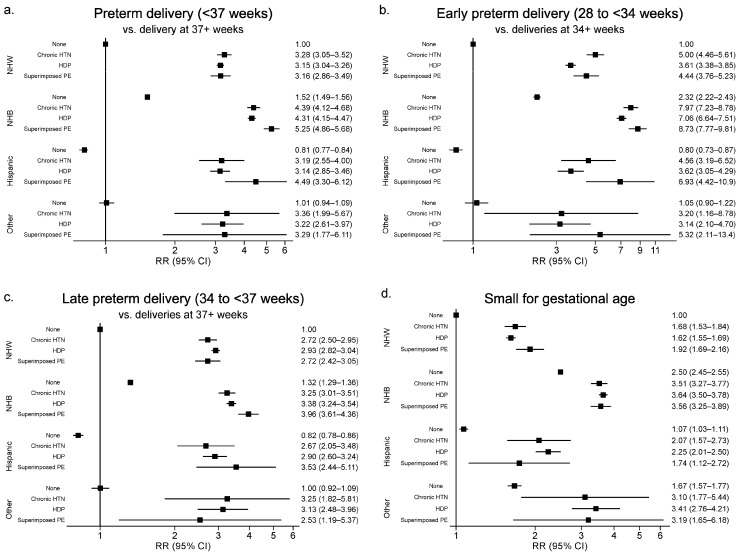
The association of hypertensive disorders of pregnancy (HDP), chronic hypertension (CHTN), and superimposed preeclampsia (superimposed PE) with (**a**) preterm delivery, (**b**) early preterm delivery, (**c**) late preterm delivery, and (**d**) small for gestational age by race/ethnicity and exposure group. NHB indicates non-Hispanic Black and NHW indicates non-Hispanic White women. The study population for preterm delivery (**a**), early preterm delivery (**b**), and small for gestational age (SGA) (**d**) was n = 666,905. The study population for late preterm delivery (**c**) was n = 650,997. All models included a significant (*p* < 0.001) interaction of exposure group and race/ethnicity. Models were adjusted for the following sociodemographic, behavioral, and clinical characteristics: maternal age (continuous), race/ethnic group, education, income (categorical), rural residence, primary payer during pregnancy, WIC eligibility during pregnancy, smoking during pregnancy, pre-pregnancy BMI (continuous), number of previous pregnancies, previous cesarean section, previous preterm delivery, and baby sex.

Models of the association of chronic hypertension, HDP or superimposed preeclampsia with PTD or SGA included a significant interaction between exposure group and race/ethnicity (*p* < 0.001). RRs for the joint effect of the hypertension exposure conditions (i.e., chronic hypertension, HDP, superimposed preeclampsia, or no hypertension) with race/ethnicity after adjustment for sociodemographic, behavioral, and clinical characteristics are shown in Figure 2. NHW women with no hypertension served as the referent group. In general, infants born to women of any race/ethnic group with any of the hypertension exposure conditions had higher risk of PTD or SGA compared with infants born to NHW women with no hypertension after adjustment for maternal age (continuous), race/ethnic group, education, income (categorical), rural residence, primary payer during pregnancy, WIC eligibility during pregnancy, smoking during pregnancy, pre-pregnancy BMI (continuous), number of previous pregnancies, previous C-section, previous PTD, and baby sex.

#### 3.2.1. Any Preterm Delivery (PTD)

Compared to NHW women with no hypertension: NHW women with chronic hypertension, HDP or superimposed preeclampsia each exhibited a three-fold increase in the risk of PTD. NHB women with no hypertension had a 52% higher risk of PTD than NHW women with no hypertension. Relative to NHW women with no hypertension, NHB women with chronic hypertension or HDP alone had a four-fold increase in risk (RR = 4.39 and RR = 4.31, respectively) and NHB women with superimposed preeclampsia showed over a five-fold increase in risk of PTD. Relative to NHW women, Hispanic women with no hypertension had ~20% lower risk of PTD (RR = 0.81). Relative to NHW women with no hypertension, Hispanic women with chronic hypertension, HDP, and superimposed preeclampsia showed elevated risks (RR = 3.19, 3.14, and 4.49, respectively). A similar pattern was seen in women of other races/ethnicities (RR = 3.36, 3.22, and 3.29, respectively). 

#### 3.2.2. Early Preterm Delivery (PTD)

Compared to NHW women with no hypertension, NHW women with chronic hypertension had five times the risk of early PTD, NHW women with HDP had lower but still elevated risk (RR = 3.61), while NHW women with superimposed preeclampsia exhibited 4.4 times the risk of early PTD. Compared to NHW women with no hypertension, NHB women with no hypertension had a 2.3 times higher risk of early PTD. Compared to NHW women with no hypertension, NHB women with chronic hypertension, HDP, or superimposed preeclampsia all had substantially elevated risks (RR = 7.97, 7.06, and 8.73, respectively). Compared to NHW women with no hypertension, Hispanic women with no hypertension had ~20% lower risk of early PTD (RR = 0.8). Compared to NHW women with no hypertension, Hispanic women with chronic hypertension, HDP, and superimposed preeclampsia had elevated risks (RR = 4.56, 3.62, and 6.93, respectively). A similar pattern was seen in women of other races/ethnicities (RR = 3.20, 3.14, and 5.32, respectively).

#### 3.2.3. Late Preterm Delivery (PTD)

With regard to late PTD, results roughly followed the same pattern as results for any PTD (<37 weeks). Relative to NHW women with no hypertension: the adjusted RR was 32% higher in NHB women, 18% lower in Hispanic women and similar in women of other race/ethnicity with no hypertension. In general, compared to NHW women with no hypertension, regardless of race/ethnicity, women with chronic hypertension, HDP and superimposed preeclampsia had higher risk of late PTD, ranging from 2.5 times the risk in women of other races/ethnicities with superimposed preeclampsia to almost four times the risk in Hispanic women with superimposed preeclampsia. 

#### 3.2.4. Small for Gestational Age (SGA)

With regard to SGA, relative to NHW women with no hypertension, the adjusted RR was 2.5 times higher in NHB women, 7% higher in Hispanic women and 67% higher in women of other race/ethnicity with no hypertension. NHW women had elevated risks in those with chronic hypertension, HDP and superimposed preeclampsia (RR = 1.68, 1.62 and 1.92, respectively) as did Hispanic women (RR = 2.07, 2.25, and 1.74, respectively) and NHB women (RR = 3.51, 3.64, and 3.56, respectively). 

#### 3.2.5. Sensitivity Analysis

When limiting the analysis to adults, the risks of infant mortality and any, early, or late preterm delivery changed by less than 10%. With regard to SGA, in general, the risk changed by less than 10% with the exception of Hispanic women with chronic hypertension and superimposed preeclampsia (decreased by 15%; RR = 1.48, 95% CI: 0.90–2.45). Results of the analyses limited to women aged 20+ years are included in Supplemental Table S2.

## 4. Discussion

In a population-based study involving statewide data for SC, infants born to women with chronic hypertension, HDP or superimposed preeclampsia were more likely to suffer adverse pregnancy outcomes (infant mortality, PTD or SGA) after adjustment for demographic and clinical characteristics compared to infants born to women without these conditions. The main study findings are summarized in Supplemental Table S3. 

The association between chronic hypertension, HDP and superimposed preeclampsia with infant mortality did not differ by race/ethnicity. After adjusting for sociodemographic, behavioral, and clinical characteristics, relative to women with no hypertension, we report maternal chronic hypertension is associated with a 48% higher risk, maternal HDP is associated with a 39% higher risk, and maternal superimposed preeclampsia is associated with a 79% higher risk of infant mortality across all race/ethnicity groups. 

In contrast, the association between chronic hypertension, HDP and superimposed preeclampsia for the PTD outcomes differed by mother’s race/ethnicity. As the baseline RR varied substantially by race/ethnicity, we evaluated the joint effect of HDP and race/ethnicity relative to NHW women with no hypertension. The risk of PTD in women with no hypertension was similar in NHW women and women of other races/ethnicities, lower in Hispanic women, and higher in NHB women. After adjusting for sociodemographic, behavioral, and clinical characteristics, relative to NHW women with no hypertension, the RR for any PTD (20 to less than 37 weeks) ranged from 3.1 to 5.3 for women with chronic hypertension, HDP and superimposed preeclampsia across all race-ethnic groups, respectively (Figure 2). Similar patterns were observed for early PTD (20 to <34 weeks), with slightly stronger RRs ranging from 3.1 to 8.7, and for late PTD (34 to <37 weeks), with slightly weaker RRs ranging from 2.5 to 3.5.

The association between chronic hypertension, HDP and superimposed preeclampsia with SGA also differed by mother’s race/ethnicity. The risks of SGA in women with no hypertension were higher in NHB (RR = 2.5), Hispanic (RR = 1.1) and the other race/ethnicity group (RR = 1.7) when compared to NHW women. After adjusting for sociodemographic, behavioral, and clinical characteristics, relative to NHW women with no hypertension, the RRs for SGA ranged from 1.6 to 1.9 in NHW women, from 3.5 to 3.6 in NHB women, from 1.7 to 2.3 in Hispanic women and from 3.1 to 3.4 in women of other races/ethnicities. When performing the sensitivity analysis, we found that the RR in adult Hispanic women decreased by 16% (RR = 1.48, 95% CI: 0.9–2.45) compared with the RR in the full dataset (RR = 1.74, 95% CI: 1.12–2.72), attenuating the risk of SGA. However, there were only 25 Hispanic women with SGA who experienced chronic hypertension with superimposed preeclampsia, and this number was reduced to 20 after removing adolescents. Thus, findings should be interpreted with caution given the small sample size and reduced power. Previous studies have shown a significant association between the presence of hypertension in any form and higher rates of stillbirth, neonatal mortality, and infant mortality as well as PTD and SGA [1,3,4,7,11,23,24,25,26]. This is consistent with our findings of an increased risk of infant mortality, PTD, and SGA in infants born to women with any HDP. 

Few studies have explored the combined relationship of chronic hypertension and superimposed preeclampsia with pregnancy outcomes. Older studies have shown that chronic hypertension was associated with adverse pregnancy outcomes, regardless of superimposed preeclampsia. [27,28]. With regard to infant mortality, our finding of similar risks of infant mortality in women with chronic hypertension, HDP or superimposed preeclampsia confirm those findings. 

The risk of infant mortality, PTD and SGA significantly differs by race/ethnicity; after adjustment for HDP and other demographic and clinical characteristics, we found that infant mortality rates continue to be higher among NHB mothers (RR = 1.43; 95% CI: 1.30–1.57) in comparison to NHW mothers as seen in other studies [17,29,30]. In 2020, infant mortality rates in both the U.S. and SC were 6.5 infant deaths per 1000 live births; however, infant mortality rates differed greatly in SC for women of White and Black/Other races with 4.5 and 10.8 infant deaths per 1000 live births, respectively [31]. NHB infant mortality rates in SC are more than twice the Healthy People 2030 objective of 5.0 infant deaths per 1000 live births [32]. 

Past studies have not been able to consider how race/ethnicity may interact with chronic hypertension, HDP, and superimposed preeclampsia [3,5,17]. We saw that in women with no hypertension the risk of PTD was 52% higher in NHB women and 19% lower in Hispanic women compared with NHW women while the risk of SGA was 2.5 times higher in NHB compared with NHW women. After controlling for the different baseline risks, most race/ethnic groups showed similarly elevated risks across chronic hypertension, HDP and superimposed preeclampsia for PTD outcomes as well as SGA. Prior studies have shown higher odds of entering pregnancy with chronic hypertension and of developing preeclampsia in NHB women [14,33]. NHB mothers have been reported to experience higher rates of having an infant born SGA or at a low birthweight [6,16]. We found that the risk of SGA was 2.5 times higher in NHB women compared with NHW women in those without hypertension. Hence, the effect of chronic hypertension, HDP or superimposed preeclampsia does not completely explain higher risk of SGA in NHB women compared to NHW women. 

### 4.1. Prevention Strategies

It may be possible to prevent HDP through treatment of hypertension and other lifestyle changes during and prior to pregnancy including physical activity, diet, and low-dose aspirin [34]. A number of risk factors have been identified for preeclampsia that are considered to be high risk (e.g., chronic conditions, past preeclampsia, multifetal pregnancy), moderate risk (e.g., demographics, pre-pregnancy obesity, family history), due to other risks (e.g., pregnancy overweight BMI, comorbidities), or due to genetic susceptibility [34,35,36]. Thus, it is essential to screen for cardiovascular disease and comorbidities both prior to and during pregnancy as possible, and to monitor and provide education to women with regard to modifiable conditions and risk factors. This is especially important for Black women and others at high-risk of poor maternal and perinatal outcomes [37,38,39]. Due to their immature physiology, pregnant adolescents may be at higher risk of pregnancy complications and adverse infant outcomes, with more frequent prenatal care recommended for assessment and management of risk factors [40].

### 4.2. Strengths and Limitations

A statewide retrospective cohort study design was used to examine differences in infant mortality, SGA, and PTD by hypertension status and racial/ethnic group. The current study was able to adjust for individual-level sociodemographic, clinical, and behavioral characteristics which could influence both the exposure and the outcomes. There are some limitations of our study including a small number of events among women with chronic hypertension and women of other race/ethnic groups that may reduce power. Other limitations of the data are the lack of information on ultrasound findings, vital signs, blood pressure measurements, medication use including antihypertensives, and biomarkers. A recent meta-analysis of antihypertensive medication use during pregnancy did not find a reduced risk of adverse perinatal outcomes [12]. While fetal growth restriction is a known cause of SGA and a primary factor in adverse infant outcomes, the lack of ultrasound findings meant that we could not reliably distinguish fetal growth restriction from constitutionally small infants using birth weight alone [41,42,43].

## 5. Conclusions

Deliveries in women with chronic hypertension, HDP or superimposed preeclampsia were more likely to result in infant mortality, PTD, and SGA than infants born to women without hypertension; baseline PTD and SGA risk differed significantly by race/ethnicity. Prevention efforts for populations at high risk of adverse pregnancy outcomes should encourage women to seek screening to determine their risk level. As chronic hypertension, HDP or superimposed preeclampsia confer higher risk of these adverse outcomes, public health interventions and clinical efforts should involve encouraging and supporting mothers to reduce modifiable cardiovascular risk factors for the prevention of morbidity and mortality. However, as these hypertensive disorders do not fully explain racial/ethnic disparities in PTD and SGA, more research to examine factors such as other social determinants of health that contribute to differences in outcomes is necessary to mitigate these disparities.

## Figures and Tables

**Table 1:** Characteristics of women by hypertensive disorders of pregnancy (HDP) and/or chronic hypertension (HTN) status ^a^.

		Total	No HTN	Chronic HTN	HDP	Superimposed Preeclampsia
		N = 666,905	N = 594,383	N = 12,180	N = 53,582	N = 6760
			(89.1%)	(1.8%)	(8.0%)	(1.0%)
Characteristic		n (%) or Mean ± SD	n (%) or Mean ± SD	n (%) or Mean ± SD	n (%) or Mean ± SD	n (%) or Mean ± SD
One year infant mortality		2426 (0.4)	2035 (0.3)	73 (0.6)	273 (0.5)	45 (0.7)
Any PTD (20 to <37 weeks)		62,215 (9.3)	47,043 (7.9)	2722 (22.3)	10,882 (20.3)	1568 (23.2)
Early PTD (20 to <34 weeks)		15,908 (2.4)	10,982 (1.8)	991 (8.1)	3362 (6.3)	573 (8.5)
Late PTD (34 to <37 weeks)		46,307 (6.9)	36,061 (6.1)	1731 (14.2)	7520 (14.0)	995 (14.7)
Small for gestational age		71,301 (10.7)	60,573 (10.2)	1667 (13.7)	8057 (15.0)	1004 (14.9)
Maternal age at delivery		26.7 ± 5.9	26.6 ± 5.9	29.0 ± 5.9	26.7 ± 6.2	29.8 ± 6.2
Race						
	Non-Hispanic White	381,325 (57.2)	344,249 (57.9)	5552 (45.6)	28,693 (53.5)	2831 (41.9)
	Non-Hispanic Black	213,516 (32.0)	182,473 (30.7)	6025 (49.5)	21,379 (39.9)	3639 (53.8)
	Hispanic	58,696 (8.8)	55,082 (9.3)	499 (4.1)	2893 (5.4)	222 (3.3)
	Other	13,368 (2.0)	12,579 (2.1)	104 (0.9)	617 (1.2)	68 (1.0)
Education						
	Less than high school	136,541 (20.5)	123,117 (20.7)	2198 (18.0)	10,280 (19.2)	946 (14.0)
	High school graduate	166,683 (25.0)	147,431 (24.8)	3244 (26.6)	14,159 (26.4)	1849 (27.4)
	Some college	160,841 (24.1)	141,764 (23.9)	3260 (26.8)	14,005 (26.1)	1812 (26.8)
	College graduate and above	202,840 (30.4)	182,071 (30.6)	3478 (28.6)	15,138 (28.3)	2153 (31.8)
Rural residence ^b^		177,769 (26.7)	159,071 (26.8)	3391 (27.8)	13,470 (25.1)	1837 (27.2)
Primary payer (during pregnancy)						
	Medicaid	338,404 (50.7)	299,558 (50.4)	6818 (56.0)	28,638 (53.4)	3390 (50.1)
	Private	253,216 (38.0)	225,554 (37.9)	4539 (37.3)	20,310 (37.9)	2813 (41.6)
	Self-pay	36,443 (5.5)	34,142 (5.7)	399 (3.3)	1735 (3.2)	167 (2.5)
	Other	33,442 (5.0)	30,311 (5.1)	348 (2.9)	2463 (4.6)	320 (4.7)
WIC eligibility during pregnancy		346,663 (52.0)	306,109 (51.5)	6680 (54.8)	30,014 (56.0)	3860 (57.1)
Smoking during pregnancy		80,396 (12.1)	72,059 (12.1)	1439 (11.8)	6176 (11.5)	722 (10.7)
Pre-pregnancy BMI		27.1 ± 6.7	26.6 ± 6.4	32.2 ± 7.9	30.3 ± 7.6	33.9 ± 7.8
Number of prior pregnancies		1.0 ± 1.2	1.0 ± 1.2	1.6 ± 1.4	0.7 ± 1.1	1.0 ± 1.3
Previous cesarean section						
	No	252,334 (37.8)	224,950 (37.8)	4659 (38.3)	20,227 (37.7)	2498 (37.0)
	Yes	46,300 (6.9)	40,599 (6.8)	2029 (16.7)	3170 (5.9)	502 (7.4)
	Unknown	368,271 (55.2)	328,834 (55.3)	5492 (45.1)	30,185 (56.3)	3760 (55.6)
Previous preterm births		19,822 (3.0)	16,681 (2.8)	1010 (8.3)	1781 (3.3)	350 (5.2)
Prenatal care as measured by R-GINDEX						
	Inadequate	118,499 (17.8)	108,481 (18.3)	1644 (13.5)	7666 (14.3)	708 (10.5)
	Intermediate	147,374 (22.1)	133,654 (22.5)	2179 (17.9)	10,456 (19.5)	1085 (16.1)
	Adequate	10,338 (1.6)	9117 (1.5)	191 (1.6)	921 (1.7)	109 (1.6)
	Intensive	187,043 (28.0)	159,513 (26.8)	4638 (38.1)	19,784 (36.9)	3108 (46.0)
	No care	6382 (1.0)	5698 (1.0)	158 (1.3)	468 (0.9)	58 (0.9)
	Missing	197,269 (29.6)	177,920 (29.9)	3370 (27.7)	14,287 (26.7)	1692 (25.0)
Pre-pregnancy diabetes or GDM		41,624 (6.2)	31,441 (5.3)	2117 (17.4)	6532 (12.2)	1534 (22.7)
Birth weight (g)		3252.2 ± 566.8	3277.8 ± 541.3	3035.6 ± 738.4	3051.2 ± 699.2	2984.0 ± 737.2
Low birth weight (<2500 g)		50,945 (7.6)	38,051 (6.4)	2239 (18.4)	9304 (17.4)	1351 (20.0)
Very low birth weight (<1500 g)		8226 (1.2)	5304 (0.9)	589 (4.8)	1979 (3.7)	354 (5.2)
Size						
	Appropriate for gestational age	536,023 (80.4)	480,925 (80.9)	9280 (76.2)	40,670 (75.9)	5148 (76.2)
	Small for gestational age	71,301 (10.7)	60,573 (10.2)	1667 (13.7)	8057 (15.0)	1004 (14.9)
	Large for gestational age	59,561 (8.9)	52,867 (8.9)	1233 (10.1)	4854 (9.1)	607 (9.0)
Male baby		340,208 (51.0)	303,134 (51.0)	6181 (50.7)	27,474 (51.3)	3419 (50.6)

PTD, preterm delivery; GDM, gestational diabetes mellitus; g, grams; HDP, hypertensive disorders of pregnancy; HTN, hypertension; No., number; R-GINDEX, Revised-Graduated Prenatal Care Utilization Index; WIC, Women, Infants and Children. ^a^ Variables with missing values included: primary payer during pregnancy (n = 5400, 0.8%), smoking during pregnancy (n = 337, 0.1%), and size for gestational age (n = 20, 0.0%)**.** ^b^ Urban/rural residence was based on rural-urban commuting area (RUCA) by zip code of residence.

**Table 2:** Results of the association of hypertensive disorders of pregnancy (HDP) and chronic hypertension with infant mortality (within one year of birth).

		Infant Mortality		
		No	Yes	
		N = 664,479 (99.6%)	N = 2426 (0.4%)	Adjusted * RR (95% CI)
Characteristic		n (%) or Mean ± SD	n (%) or Mean ± SD	
No HTN		592,348 (89.1)	2035 (83.9)	referent
Chronic HTN		12,107 (1.8)	73 (3.0)	1.48 (1.16–1.88)
HDP		53,309 (8.0)	273 (11.3)	1.39 (1.21–1.58)
Superimposed preeclampsia		6715 (1.0)	45 (1.9)	1.79 (1.31–2.43)
Maternal age at delivery		26.7 ± 5.9	25.4 ± 6.0	0.98 (0.97–0.99)
Race				
	Non-Hispanic White	380,150 (57.2)	1175 (48.4)	referent
	Non-Hispanic Black	212,425 (32.0)	1091 (45.0)	1.43 (1.30–1.57)
	Hispanic	58,559 (8.8)	137 (5.6)	0.67 (0.54–0.82)
	Other	13,345 (2.0)	23 (0.9)	0.75 (0.49–1.13)
Education				
	Less than high school	135,854 (20.4)	687 (28.3)	1.53 (1.30–1.80)
	High school graduate	165,949 (25.0)	734 (30.3)	1.41 (1.21–1.63)
	Some college	160,251 (24.1)	590 (24.3)	1.29 (1.12–1.49)
	College graduate and above	202,425 (30.5)	415 (17.1)	referent
Urban/rural (based on RUCA by zipcode of residence)				
	Urban	487,429 (73.4)	1707 (70.4)	referent
	Rural	177,050 (26.6)	719 (29.6)	0.96 (0.87–1.06)
Primary payer (during pregnancy) from birth records				
	Medicaid	336,808 (50.7)	1596 (65.8)	1.38 (1.22–1.56)
	Private	252,644 (38.0)	572 (23.6)	referent
	Self-pay	36,313 (5.5)	130 (5.4)	1.58 (1.27–1.98)
	Other	33,337 (5.0)	105 (4.3)	1.34 (1.08–1.66)
WIC eligibility during pregnancy				
	No	308,691 (46.5)	935 (38.5)	referent
	Yes	345,214 (52.0)	1449 (59.7)	0.81 (0.73–0.89)
Smoking during pregnancy				
	No	584,301 (87.9)	1871 (77.1)	referent
	Yes	79,843 (12.0)	553 (22.8)	1.88 (1.69–2.09)
Pre-pregnancy BMI		27.1 ± 6.7	28.1 ± 7.3	1.01 (1.01–1.02)
Number of prior pregnancies		1.0 ± 1.2	1.1 ± 1.3	1.06 (1.02–1.10)
Previous cesarean section				
	No	251,485 (37.8)	849 (35.0)	referent
	Yes	46,147 (6.9)	153 (6.3)	0.87 (0.73– 1.04)
	Unknown	366,847 (55.2)	1424 (58.7)	1.07 (0.98–1.17)
Previous preterm births				
	No	644,818 (97.0)	2265 (93.4)	referent
	Yes	19,661 (3.0)	161 (6.6)	1.96 (1.66–2.33)
Sex of baby				
	Male	338,796 (51.0)	1412 (58.2)	referent
	Female	325,683 (49.0)	1014 (41.8)	0.75 (0.69–0.81)

* Adjusted for mother’s race, education, rural residence, primary payer, WIC eligibility, smoking during pregnancy, pre-pregnancy BMI, number of prior pregnancies, previous cesarean section, previous preterm birth and baby sex.

## Data Availability

For confidentiality reasons authors are not allowed to share the data.

## Supplementary Materials

The following supporting information can be downloaded at: https://www.mdpi.com/article/10.3390/healthcare12050597/s1, Table S1: Diagnoses for the exposure, covariates, and outcomes of interest based on hospitalization and emergency department International Classification of Diseases, Ninth and Tenth Revision, Clinical Modification (ICD-9-CM) and (ICD-10-CM) codes or as reported on birth certificates or death certificates; Table S2. Sensitivity analysis of the change in risk estimates for infant outcomes when data is limited to adults aged 20 and over; Table S3. Summary of main study findings.
