# Supplementary material for: The Association of Hypertensive Disorders of Pregnancy with Infant Mortality, Preterm Delivery, and Small for Gestational Age
Source: Healthcare (Basel). 2024 Mar 6;12(5):597. doi: 10.3390/healthcare12050597 (PMC10931061; doi:10.3390/healthcare12050597)
Supplement: Supplementary file 1 [file healthcare-12-00597-s001.zip › Supplement Table S1.pdf]

Supplemental Table S1. Diagnoses for the exposure, covariates, and outcomes of interest based on hospitalization and emergency department encounters coded using the International Classification of Diseases, Ninth and Tenth Revision, Clinical Modification (ICD-9-CM) and (ICD-10-CM) codes or as reported on birth certificates or death certificates

DIAGNOSIS	ICD-9-CM CODE	ICD-10-CM CODE
Outcome of delivery (birth) *	V27.x, V30.x, 640-649, 650-659, 660-669	Z37.x
Single liveborn; single live birth *	V27.0, V30.0	Z37.0
Kidney transplant	procedure code: 55.6, V42.0	Z94.0
**Hypertensive disorders of pregnancy (HDP)**		
Gestational hypertension, Pre-eclampsia, Eclampsia, Pre-existing hypertension with pre-eclampsia	642.3, 642.4-642.5, 642.6, 642.7	O11.x, O13.x, O14.x, O15.x, O.16.x
Gestational hypertension (birth certificate)	--	--
**Pre-pregnancy hypertension**		
Benign essential hypertension complicating pregnancy, childbirth, and the puerperium; Hypertension secondary to renal disease complicating pregnancy, childbirth, and the puerperium; Other pre-existing hypertension complicating pregnancy, childbirth, and the puerperium; Pre-existing essential hypertension complicating pregnancy, childbirth, and the puerperium, Pre-existing hypertensive heart disease complicating pregnancy, childbirth and the puerperium	642.0-642.2	O10.0
Pre-pregnancy hypertension (birth certificate)	--	--
**Pre-pregnancy hypertension with superimposed HDP †**		
Pre-eclampsia or eclampsia superimposed on pre-existing hypertension; Pre-existing hypertension with pre-eclampsia	642.7	O11.x, O13.x, O14.x, O15.x

* Obtained from the mother's hospitalization/emergency department visit record.

† Also defined by a combination of the above diagnosis codes for hypertensive disorder of pregnancy and pre-pregnancy hypertension.
