# Supplementary material for: The Association of Hypertensive Disorders of Pregnancy with Infant Mortality, Preterm Delivery, and Small for Gestational Age
Source: Healthcare (Basel). 2024 Mar 6;12(5):597. doi: 10.3390/healthcare12050597 (PMC10931061; doi:10.3390/healthcare12050597)
Supplement: Supplementary file 1 [file healthcare-12-00597-s001.zip › Supplemental Table S2 change in risk.pdf]

Supplemental Table S2. Sensitivity analysis of the change in risk estimates for infant outcomes when data is limited to adults aged 20 and over.

	Full dataset			Limited to adults (20+ years)			
	**n**	**RR (95% CI)**		**n**	**RR (95% CI)**		**% change**
**Infant Mortality**							
Neither pre-pregnancy hypertension nor HDP	2,035	referent		1,680	referent		
Pre-pregnancy hypertension	73	1.48	(1.16- 1.88)	69	1.49	(1.16- 1.92)	0.8%
HDP	273	1.39	(1.21- 1.58)	233	1.44	(1.25- 1.67)	4.1%
Pre-pregnancy hypertension with superimposed HDP	45	1.79	(1.31- 2.43)	42	1.77	(1.28- 2.43)	-1.1%
**Preterm delivery (<37 weeks)**							
**Non-Hispanic White**							
Neither pre-pregnancy hypertension nor HDP	23,865	referent		21,280	referent		
Pre-pregnancy hypertension	1,077	3.22	(3.00- 3.46)	1,010	3.14	(3.00- 3.46)	*-2.5%*
HDP	5,180	3.09	(2.98- 3.20)	4,689	3.16	(2.98- 3.20)	*2.4%*
Pre-pregnancy hypertension with superimposed HDP	510	3.07	(2.78- 3.40)	485	3.01	(2.78- 3.40)	*-2.2%*
**Non-Hispanic Black**							
Neither pre-pregnancy hypertension nor HDP	18,743	referent		15,684	referent		
Pre-pregnancy hypertension	1,518	2.95	(2.77- 3.15)	1,451	2.97	(2.79- 3.18)	*0.8%*
HDP	4,995	2.89	(2.79- 3.00)	4,273	2.98	(2.86- 3.11)	*3.2%*
Pre-pregnancy hypertension with superimposed HDP	987	3.53	(3.26- 3.82)	934	3.53	(3.25- 3.83)	*0.1%*
**Hispanic**							
Neither pre-pregnancy hypertension nor HDP	3,553	referent		3,112	referent		
Pre-pregnancy hypertension	105	3.82	(3.04- 4.80)	98	3.66	(2.89- 4.62)	*-4.3%*
HDP	591	3.75	(3.39- 4.16)	532	3.91	(3.51- 4.35)	*4.0%*
Pre-pregnancy hypertension with superimposed HDP	60	5.19	(3.80- 7.08)	55	4.86	(3.52- 6.72)	*-6.2%*
**Other**							
Neither pre-pregnancy hypertension nor HDP	882	referent		835	referent		
Pre-pregnancy hypertension	22	2.98	(1.75- 5.08)	22	3.10	(1.84- 5.22)	*4.1%*
HDP	116	2.94	(2.34- 3.69)	114	3.18	(2.53- 3.99)	*8.3%*
Pre-pregnancy hypertension with superimposed HDP	14	2.78	(1.48- 5.22)	14	2.90	(1.54- 5.47)	*4.5%*
**Early preterm delivery (28 to <34 weeks)**							
**Non-Hispanic White**							
Neither pre-pregnancy hypertension nor HDP	4,619	referent		3,983	referent		
Pre-pregnancy hypertension	355	4.95	(4.40- 5.57)	330	4.90	(4.34- 5.53)	*-1.0%*
HDP	1,339	3.56	(3.34- 3.80)	1,208	3.74	(3.49- 4.00)	*4.9%*
Pre-pregnancy hypertension with superimposed HDP	164	4.46	(3.78- 5.28)	152	4.29	(3.60- 5.10)	*-4.0%*
**Non-Hispanic Black**							
Neither pre-pregnancy hypertension nor HDP	5,443	referent		4,540	referent		
Pre-pregnancy hypertension	597	3.48	(3.16- 3.82)	571	3.53	(3.21- 3.89)	*1.7%*

HDP	1,831	3.06	(2.89-3.24)	1,610	3.22	(3.03-3.43)	*5.4%*
Pre-pregnancy hypertension with superimposed HDP	380	3.69	(3.28-4.14)	360	3.66	(3.25-4.13)	*-0.7%*
**Hispanic**							
Neither pre-pregnancy hypertension nor HDP	735		referent	632		referent	
Pre-pregnancy hypertension	34	5.33	(3.69-7.70)	32	5.06	(3.46-7.40)	*-5.1%*
HDP	165	4.28	(3.56-5.15)	151	4.48	(3.70-5.43)	*4.8%*
Pre-pregnancy hypertension with superimposed HDP	23	7.73	(4.86-12.31)	21	6.97	(4.28-11.33)	*-9.9%*
**Other**							
Neither pre-pregnancy hypertension nor HDP	185		referent	170		referent	
Pre-pregnancy hypertension	5	2.72	(0.95-7.74)	5	2.75	(0.97-7.80)	*1.1%*
HDP	27	2.97	(1.91-4.64)	26	3.08	(1.96-4.85)	*3.6%*
Pre-pregnancy hypertension with superimposed HDP	6	4.97	(1.87-13.18)	6	5.35	(2.01-14.21)	*7.6%*
**Late preterm delivery (34 to <37 weeks)**							
**Non-Hispanic White**							
Neither pre-pregnancy hypertension nor HDP	19,246		referent	17,297		referent	
Pre-pregnancy hypertension	722	2.64	(2.43-2.87)	680	2.60	(2.39-2.83)	*-1.4%*
HDP	3,841	2.86	(2.75-2.98)	3,481	2.92	(2.80-3.04)	*2.1%*
Pre-pregnancy hypertension with superimposed HDP	346	2.61	(2.32-2.94)	333	2.59	(2.30-2.92)	*-0.9%*
**Non-Hispanic Black**							
Neither pre-pregnancy hypertension nor HDP	13,300		referent	11,144		referent	
Pre-pregnancy hypertension	921	2.50	(2.32-2.71)	880	2.54	(2.35-2.75)	*1.6%*
HDP	3,164	2.65	(2.53-2.76)	2,663	2.68	(2.55-2.81)	*1.4%*
Pre-pregnancy hypertension with superimposed HDP	604	3.15	(2.86-3.46)	574	3.16	(2.86-3.48)	*0.2%*
**Hispanic**							
Neither pre-pregnancy hypertension nor HDP	2,818		referent	2,480		referent	
Pre-pregnancy hypertension	71	3.21	(2.46-4.19)	66	3.08	(2.34-4.06)	*-4.1%*
HDP	426	3.45	(3.07-3.88)	381	3.57	(3.16-4.04)	*3.5%*
Pre-pregnancy hypertension with superimposed HDP	37	4.10	(2.83-5.95)	34	3.91	(2.66-5.76)	*-4.7%*
**Other**							
Neither pre-pregnancy hypertension nor HDP	697		referent	665		referent	
Pre-pregnancy hypertension	17	2.94	(1.63-5.31)	17	3.09	(1.74-5.48)	*5.0%*
HDP	89	2.82	(2.19-3.63)	88	3.08	(2.40-3.97)	*9.5%*
Pre-pregnancy hypertension with superimposed HDP	8	2.07	(0.96-4.47)	8	2.15	(1.00-4.66)	*3.9%*
**Small for gestational age**							
**Non-Hispanic White**							
Neither pre-pregnancy hypertension nor HDP	25,374		referent	22,057		referent	
Pre-pregnancy hypertension	549	1.69	(1.54-1.86)	520	1.75	(1.59-1.93)	*3.6%*
HDP	3,076	1.64	(1.57-1.71)	2,685	1.69	(1.62-1.77)	*3.2%*

Pre-pregnancy hypertension with superimposed HDP	316	1.93	(1.70- 2.18)	304	2.03	(1.79- 2.30)	*5.6%*
**Non-Hispanic Black**							
Neither pre-pregnancy hypertension nor HDP	29,420		referent	23,298		referent	
Pre-pregnancy hypertension	1,039	1.39	(1.29- 1.49)	972	1.40	(1.30- 1.51)	*0.9%*
HDP	4,442	1.45	(1.40- 1.50)	3,540	1.47	(1.41- 1.53)	*1.6%*
Pre-pregnancy hypertension with superimposed HDP	652	1.41	(1.29- 1.54)	603	1.43	(1.30- 1.56)	*1.2%*
**Hispanic**							
Neither pre-pregnancy hypertension nor HDP	4,360		referent	3,636		referent	
Pre-pregnancy hypertension	62	2.08	(1.57- 2.76)	57	2.22	(1.67- 2.95)	*6.6%*
HDP	420	2.13	(1.90- 2.39)	357	2.22	(1.97- 2.51)	*4.2%*
Pre-pregnancy hypertension with superimposed HDP	25	1.74	(1.12- 2.72)	20	1.48	(0.89- 2.44)	*-15.1%*
**Other**							
Neither pre-pregnancy hypertension nor HDP	1,419		referent	1,353		referent	
Pre-pregnancy hypertension	17	2.03	(1.15- 3.58)	17	1.89	(1.07- 3.34)	*-6.6%*
HDP	119	2.13	(1.70- 2.66)	113	2.12	(1.69- 2.65)	*-0.5%*
Pre-pregnancy hypertension with superimposed HDP	11	2.20	(1.13- 4.29)	10	1.98	(0.99- 3.97)	*-9.8%*
