# Supplementary material for: The Association of Hypertensive Disorders of Pregnancy with Infant Mortality, Preterm Delivery, and Small for Gestational Age
Source: Healthcare (Basel). 2024 Mar 6;12(5):597. doi: 10.3390/healthcare12050597 (PMC10931061; doi:10.3390/healthcare12050597)
Supplement: Supplementary file 1 [file healthcare-12-00597-s001.zip › Supplemental Table S3 overall study findings.pdf]

Supplemental Table S3. Summary of main study findings

	n	RR (95% CI)		p for interaction of case/race
**Infant Mortality**				
Neither pre-pregnancy hypertension nor HDP	2,035	referent		0.62
Pre-pregnancy hypertension	73	1.48	(1.16- 1.88)	
HDP	273	1.39	(1.21- 1.58)	
Pre-pregnancy hypertension with superimposed HDP	45	1.79	(1.31- 2.43)	
**Preterm delivery (<37 weeks)**				<0.001
**Non-Hispanic White**				
Neither pre-pregnancy hypertension nor HDP	23,865	referent		
Pre-pregnancy hypertension	1,077	3.22	(3.00- 3.46)	
HDP	5,180	3.09	(2.98- 3.20)	
Pre-pregnancy hypertension with superimposed HDP	510	3.07	(2.78- 3.40)	
**Non-Hispanic Black**				
Neither pre-pregnancy hypertension nor HDP	18,743	1.52	(1.49- 1.56)	
Pre-pregnancy hypertension	1,518	4.39	(4.12- 4.68)	
HDP	4,995	4.31	(4.15- 4.47)	
Pre-pregnancy hypertension with superimposed HDP	987	5.25	(4.86- 5.68)	
**Hispanic**				
Neither pre-pregnancy hypertension nor HDP	3,553	0.81	(0.77- 0.84)	
Pre-pregnancy hypertension	105	3.19	(2.55- 4.00)	
HDP	591	3.14	(2.85- 3.46)	
Pre-pregnancy hypertension with superimposed HDP	60	4.49	(3.30- 6.12)	
**Other**				
Neither pre-pregnancy hypertension nor HDP	882	1.01	(0.94- 1.09)	
Pre-pregnancy hypertension	22	3.36	(1.99- 5.67)	
HDP	116	3.22	(2.61- 3.97)	
Pre-pregnancy hypertension with superimposed HDP	14	3.29	(1.77- 6.11)	
**Early preterm delivery (28 to <34 weeks)**				<0.001
**Non-Hispanic White**				
Neither pre-pregnancy hypertension nor HDP	4,619			
Pre-pregnancy hypertension	355	4.95	(4.40- 5.57)	
HDP	1,339	3.56	(3.34- 3.80)	
Pre-pregnancy hypertension with superimposed HDP	164	4.46	(3.78- 5.28)	
**Non-Hispanic Black**				
Neither pre-pregnancy hypertension nor HDP	5,443	2.32	(2.22- 2.43)	
Pre-pregnancy hypertension	597	7.97	(7.23- 8.78)	
HDP	1,831	7.06	(6.64- 7.51)	
Pre-pregnancy hypertension with superimposed HDP	380	8.73	(7.77- 9.81)	
**Hispanic**				
Neither pre-pregnancy hypertension nor HDP	735	0.80	(0.73- 0.87)	
Pre-pregnancy hypertension	34	4.56	(3.19- 6.52)	
HDP	165	3.62	(3.05- 4.29)	
Pre-pregnancy hypertension with superimposed HDP	23	6.93	(4.42- 10.89)	
**Other**				

Neither pre-pregnancy hypertension nor HDP	185	1.05	(0.90-1.22)	
Pre-pregnancy hypertension	5	3.20	(1.16-8.78)	
HDP	27	3.14	(2.10-4.70)	
Pre-pregnancy hypertension with superimposed HDP	6	5.32	(2.11-13.41)	
**Late preterm delivery (34 to <37 weeks)**				<0.001
**Non-Hispanic White**				
Neither pre-pregnancy hypertension nor HDP	19,246		referent	
Pre-pregnancy hypertension	722	2.64	(2.43-2.87)	
HDP	3,841	2.86	(2.75-2.98)	
Pre-pregnancy hypertension with superimposed HDP	346	2.61	(2.32-2.94)	
**Non-Hispanic Black**				
Neither pre-pregnancy hypertension nor HDP	13,300	1.32	(1.29-1.36)	
Pre-pregnancy hypertension	921	3.25	(3.01-3.51)	
HDP	3,164	3.38	(3.24-3.54)	
Pre-pregnancy hypertension with superimposed HDP	604	3.96	(3.61-4.36)	
**Hispanic**				
Neither pre-pregnancy hypertension nor HDP	2,818	0.82	(0.78-0.86)	
Pre-pregnancy hypertension	71	2.67	(2.05-3.48)	
HDP	426	2.90	(2.60-3.24)	
Pre-pregnancy hypertension with superimposed HDP	37	3.53	(2.44-5.11)	
**Other**				
Neither pre-pregnancy hypertension nor HDP	697	1.00	(0.92-1.09)	
Pre-pregnancy hypertension	17	3.25	(1.82-5.81)	
HDP	89	3.13	(2.48-3.96)	
Pre-pregnancy hypertension with superimposed HDP	8	2.53	(1.19-5.37)	
**Small for gestational age**				<0.001
**Non-Hispanic White**				
Neither pre-pregnancy hypertension nor HDP	25,374		referent	
Pre-pregnancy hypertension	549	1.68	(1.53-1.84)	
HDP	3,076	1.62	(1.55-1.69)	
Pre-pregnancy hypertension with superimposed HDP	316	1.92	(1.69-2.16)	
**Non-Hispanic Black**				
Neither pre-pregnancy hypertension nor HDP	29,420	2.50	(2.45-2.55)	
Pre-pregnancy hypertension	1,039	3.51	(3.27-3.77)	
HDP	4,442	3.64	(3.50-3.78)	
Pre-pregnancy hypertension with superimposed HDP	652	3.56	(3.25-3.89)	
**Hispanic**				
Neither pre-pregnancy hypertension nor HDP	4,360	1.07	(1.03-1.11)	
Pre-pregnancy hypertension	62	2.07	(1.57-2.73)	
HDP	420	2.25	(2.01-2.50)	
Pre-pregnancy hypertension with superimposed HDP	25	1.74	(1.12-2.72)	
**Other**				
Neither pre-pregnancy hypertension nor HDP	1,419	1.67	(1.57-1.77)	
Pre-pregnancy hypertension	17	3.10	(1.77-5.44)	
HDP	119	3.41	(2.76-4.21)	
Pre-pregnancy hypertension with superimposed HDP	11	3.19	(1.65-6.18)	
